# Neuroprotective Effect of Gallocatechin Gallate on Glutamate-Induced Oxidative Stress in Hippocampal HT22 Cells

**DOI:** 10.3390/molecules26051387

**Published:** 2021-03-04

**Authors:** Do Hwi Park, Jun Yeon Park, Ki Sung Kang, Gwi Seo Hwang

**Affiliations:** 1 College of Korean Medicine, Gachon University, Seongnam 13120, Korea; parkdo@gc.gachon.ac.kr; 2 Department of Food Science and Biotechnology, Kyonggi University, Suwon 16227, Korea; rhemf@kgu.ac.kr

**Keywords:** gallocatechin gallate, ROS, HT22, Ca^2+^, antioxidant

## Abstract

Oxidative stress leads to protein degeneration or mitochondrial dysfunction, causing neuronal cell death. Glutamate is a neurotransmitter that nerve cells use to send signals. However, the excess accumulation of glutamate can cause excitotoxicity in the central nervous system. In this study, we deciphered the molecular mechanism of catechin-mediated neuroprotective effect on glutamate-induced oxidative stress in mouse hippocampal neuronal HT22 cells. Cellular antioxidant activity was determined using the 1,1-diphenyl-picryl hydrazyl (DPPH) assay and 2′,7′-dichlorodihydrofluorescein diacetate (DCFDA) staining. Furthermore, the levels of intracellular calcium (Ca^2+^) as well as nuclear condensation and protein expression related to neuronal damage were assessed. All five catechins (epigallocatechin gallate, gallocatechin gallate (GCG), gallocatechin, epicatechin gallate, and epicatechin) showed strong antioxidant effects. Among them, GCG exhibited the highest neuroprotective effect against glutamate excitotoxicity and was used for further mechanistic studies. The glutamate-induced increase in intracellular Ca^2+^ was reduced after GCG treatment. Moreover, GCG reduced nuclear condensation and the phosphorylation of extracellular signal-regulated kinase (ERK) and c-Jun N-terminal kinases (JNK) involved in cell death. The neuroprotective effect of GCG against glutamate-induced oxidative stress in HT22 cells was attributed to the reduction in intracellular free radicals and Ca^2+^ influx and also the inhibition of phosphorylation of ERK and JNK. Furthermore, the antioxidant effect of GCG was found to be likely due to the inhibition of phosphorylation of ERK and JNK that led to the effective suppression of neurocytotoxicity caused by glutamate in HT22 cells.

## 1. Introduction

The incidence of degenerative brain diseases increases with age, and encephalopathy is a serious health problem in the Korean population. Degenerative brain diseases caused by both genetic and environmental factors, such as oxidative stress, are known causes of neuronal cell death due to neurodegeneration and aging, and studies are being actively pursued to determine their exact cause [1,2,3]. Glutamate is a well-known excitatory neurotransmitter, which plays an important role in the brain. However, excessive glutamate secretion outside neurons can induce neuronal cell death and is associated with various brain diseases, including neurotoxicity, ischemic stroke, Alzheimer’s disease, and Parkinson’s disease [1]. Excess glutamate increases the production of reactive oxygen species (ROS) consists of both radical and non-radical. ROS are free radicals produced during oxidative stress and are the leading cause of diseases of the brain and nervous system [4,5].

Recently, natural products and phytochemical are attractable for their neuroprotective potential medicated by antioxidant effects. They can significantly ameliorate the symptoms related to neurodegenerative diseases caused by ROS. Therefore, it is reasonable to investigate antioxidant-rich plants for the treatment of oxidative neurodegeneration.

The presence of antioxidants, such as acetyl cysteine or *N*-flavonoids, prevents neuronal cell death caused by ROS [6,7]. The use of natural antioxidants for eliminating free radicals and maintaining homeostasis contributes to alleviating neurological dysfunction [7]. It has been also shown that grape seed extracts possess high contents of flavonoids and showed neuroprotective effect in ischemic brain injury [8].

Catechins are polyphenolic compounds present in the green tea (young leaves) extract of Camellia (*Camellia sinensis* L. arachnid) and have various beneficial effects, such as antioxidant and cardioprotective properties [9]. Recently, there have been many studies on the effects of green tea extracts, catechin, and epigallocatechin gallate (EGCG) on nerve cells [9,10,11,12], and the primary focus of these studies has been the neuroprotective effect of EGCG. In this study, we compared the neuroprotective effect of five catechins (EGCG, gallocatechin gallate (GCG), gallocatechin (GC), epicatechin gallate (ECG), and epicatechin (EC)) and investigated the molecular mechanism of neuroprotective effect of GCG in glutamate excitotoxicity. EGCG exerted a strong neuroprotective effect but caused neurocytotoxicity at high concentrations. We preferred GCG over EGCG for the study as it does not cause neurocytotoxicity even at high concentrations and also exhibits neuroprotective effects. Although EGCG and GCG possess similar chemical structures, they showed a significant difference in neurocytotoxicity. The purpose of this study was to identify the molecular mechanism of neuroprotective effect of GCG in glutamate excitotoxicity.

## 2. Results

### 2.1. Antioxidant and Neuroprotective Effects of Catechins

#### In Vitro Antioxidant Effect of Catechins

**Figure 1:**
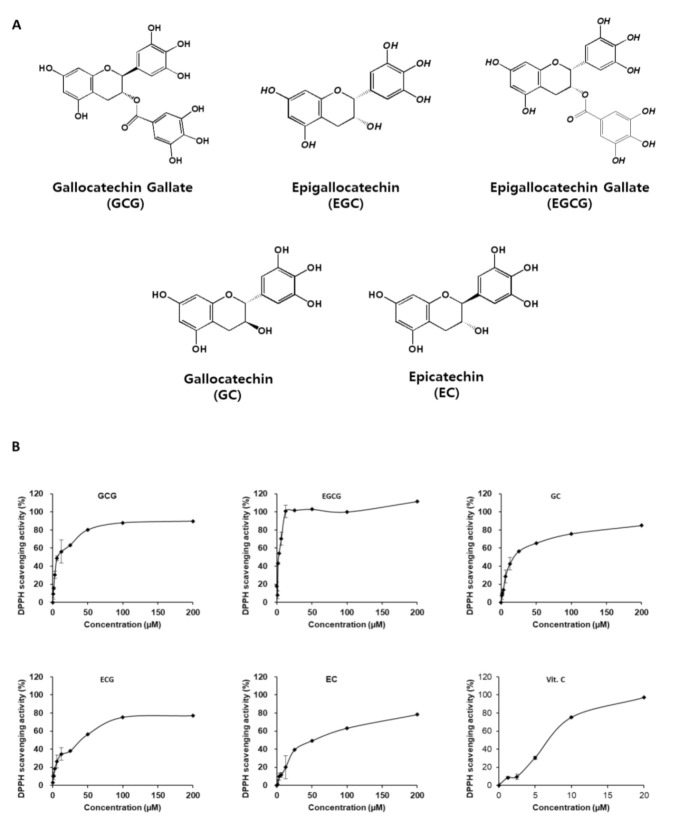
Comparison of the antioxidant effect of the five catechins. (**A**) Chemical structures of five catechins used in the study. (**B**) Comparison of DPPH radical scavenging activity of catechins (GCG, EGCG, GC, ECG, and EC); Vitamin C (Vit.C) was used as a positive control. DPPH radical scavenging activity assay was used to assess the antioxidant potential of catechins.

In general, catechins mainly include GCG, ECG, EGCG, GC, EC, and their stereoisomers, with compositions similar to each other (Figure 1A). All catechins showed 1,1-diphenyl-2-picryl-hydrazyl (DPPH) scavenging activity in a concentration-dependent manner (Figure 1B). All five catechins were evaluated for their antioxidant activity, and their IC_50_ values were as follows: GCG (IC_50_ = 7.29), EGCG (IC_50_ = 2.52), GC (IC_50_ = 19.27), ECG (IC_50_ = 41.4), and EC (IC_50_ = 52.17); vitamin C (Vit.C) (IC_50_ = 7.18) was used as a positive control. EGCG showed better activity and GCG showed similar activity when compared to Vit.C, respectively. Catechins exhibited strong DPPH scavenging potential.

### 2.2. Neuroprotective Effects of Catechins against Glutamate-Induced Apoptosis in HT22 Cells

**Figure 2:**
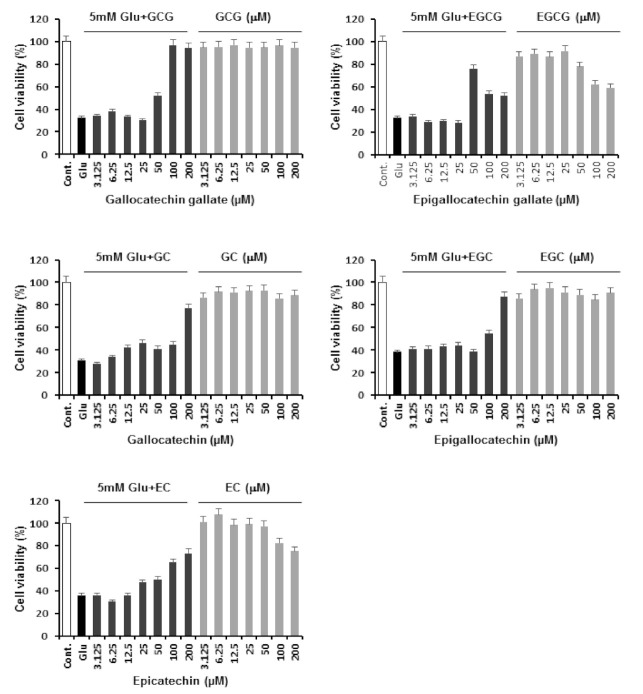
Neuroprotective effects of catechins against glutamate-induced HT22 cell death. Cell viability was measured using a CyTox assay kit 24 h after treatment with 5 mM glutamate with or without catechins. Bars denote the percentage of cell viability (compared to glutamate-treated cells).

As shown in Figure 2, in the glutamate-treated group, the cell survival rate was lowered by 60% when compared with the control group. All five catechins that were assessed showed neuroprotective effects. Among them, EGCG showed the highest neuroprotective effect at a low concentration of 50 μM. However, EGCG showed neurocytotoxicity at 100 μM and 200 μM when used without glutamate. On the contrary, GCG showed a concentration-dependent increase in cell viability from 55% at 50 µM to 96% at 100 μM, and GCG did not show neurocytotoxicity when used alone (Table 1). Therefore, it was selected for further mechanistic studies.

### 2.3. Effect of GCG on ROS Production by Glutamate in HT22 Cells

**Figure 3:**
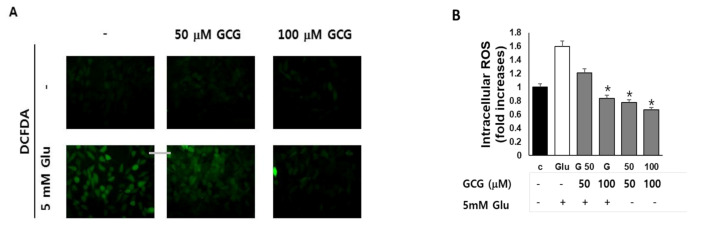
Inhibitory effect of GCG on ROS production induced by glutamate in HT22 cells. (**A**) Cells were treated with 5 mM glutamate in the presence or absence of 50 or 100 μM GCG for 8 h and stained with DCFDA. Fluorescence images of DCF were obtained using fluorescence microscopy. Scale bar, 20 μm. (**B**) Bars denote the fold increase in fluorescent intensity of DCF compared to control cells (mean ± S.E.M, * *p* < 0.001 when compared with glutamate-treated cells).

We measured the effect of GCG on intracellular ROS accumulation by glutamate in HT22 cells. Images obtained using microscopy show that GCG inhibits the ROS increase induced by glutamate in HT22 cells (Figure 3A). After 8 h of glutamate treatment, ROS increased by 1.6-fold in HT22 cells compared to those in the untreated group, and GCG pretreatment reduced the accumulation of mitochondrial ROS induced by glutamate to the level of the untreated group (Figure 3B). However, GCG did not affect mitochondrial ROS content when used alone, indicating that GCG inhibited ROS accumulation induced by glutamate.

### 2.4. Effect of GCG on Glutamate-Induced Ca^2+^ Accumulation in HT22 Cells

**Figure 4:**
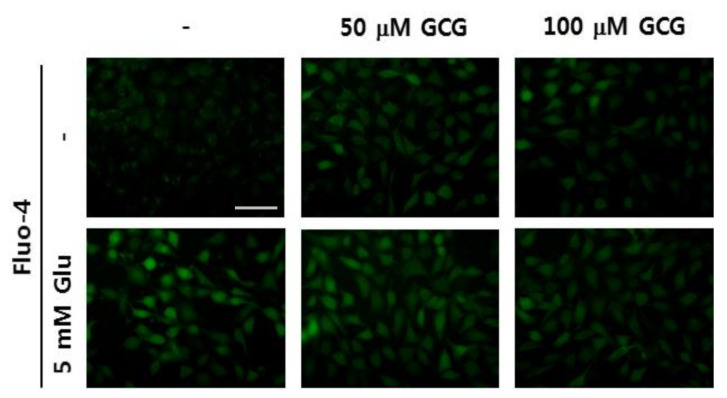
Inhibitory effect of GCG on glutamate-induced Ca^2+^ accumulation in HT22 cells. Cells were treated with 5 mM glutamate in the presence or absence of 50 or 100 μM GCG for 8 h and stained with Fluo-4. Fluorescence images of Fluo-4 were obtained using fluorescence microscopy. Scale bar, 20 μm.

Representative fluorescence images show that Fluo-4 positive cells significantly increase after glutamate treatment, whereas GCG treatment inhibits this effect (Figure 4).

### 2.5. Effect of GCG on Glutamate-Induced Nuclear Condensation in HT22 Cells

**Figure 5:**
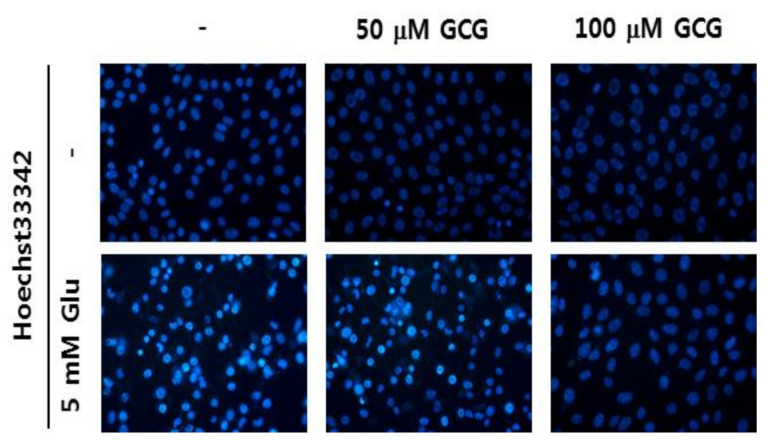
Effect of GCG on nuclear condensation. Cells were treated with 5 mM glutamate in the absence or presence of 50 or 100 μM GCG for 10 h and stained with 2 μM Hoechst 33342. Images obtained using microscopy indicate a decrease in chromatin condensation after treatment with GCG. Scale bar, 20 μm.

Figure 5 shows the nuclear condensation of HT22 cells after glutamate treatment. However, in the group treated with 100 μM of GCG, nuclear condensation hardly occurred. This result suggested that GCG inhibited glutamate-induced nuclear condensation and had anti-apoptotic activity.

### 2.6. Effect of GCG on Glutamate-Induced Phosphorylation of MAPKs in HT22 Cells

**Figure 6:**
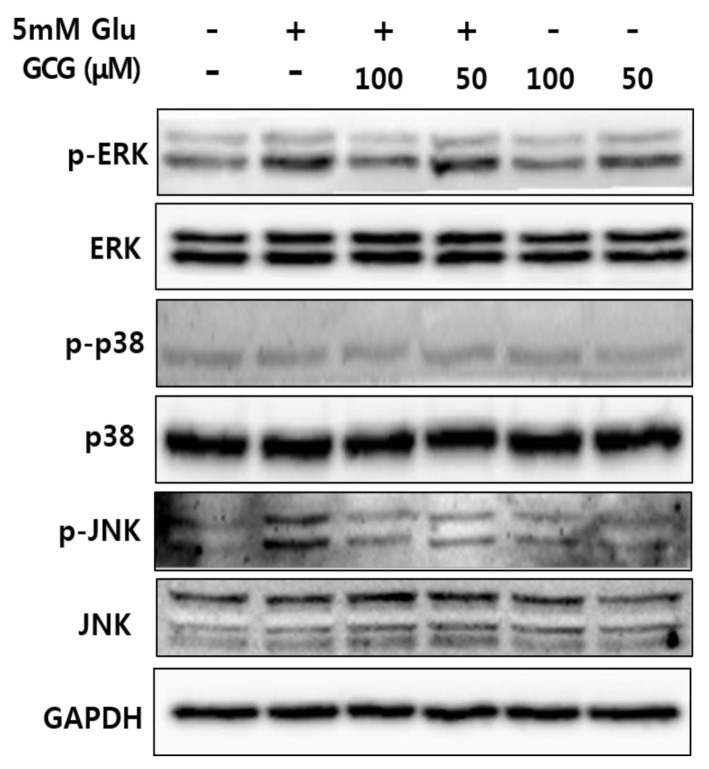
Effect of GCG on phosphorylation of mitogen-activated protein kinase (MAPK) induced by glutamate in HT22 cells. HT22 cells were treated with 5 mM glutamate with or without 50 or 100 µM GCG for 8 h. Western blot analysis was performed using specific antibodies for p38, p-p38, ERK, p-ERK, JNK, p-JNK, and GAPDH.

MAPK activation is responsible for cell death. In addition, intracellular ROS inhibition has been reported to inhibit MAPK phosphorylation and apoptosis induced by hydrogen peroxide activation, indicating the involvement of ROS-mediated phosphorylation of MAPK in neuronal cell death [13,14,15]. Therefore, we investigated whether GCG could block MAPK activation. We found that glutamate increased phosphorylation of ERK and JNK, but GCG reduced glutamate-induced phosphorylation of MAPK in a concentration-dependent manner (Figure 6). These results suggested that the inhibition of p-ERK and p-JNK was the molecular mechanism of GCG-mediated neuroprotective effect on glutamate-induced HT22 cell death.

## 3. Discussion

Glutamate is a well-known excitatory neurotransmitter present in the brain. However, excessive glutamate secretion outside the neurons leads to neuronal death, which is a major cause of degenerative brain diseases. Excess glutamate outside neurons induces excitotoxicity and causes oxidative stress, leading to early necrotic cell death and subsequent apoptotic cell death [16]. In particular, oxidative stress is a well-known factor in neuronal death. It is closely related to aging and neurodegenerative diseases [17]. The high intracellular Ca^2+^ influx caused by glutamate damages mitochondria and generates ROS from the mitochondrial electron transport system, thereby, leading to cell death. Neuronal cells are very vulnerable to oxidative stress [3,18,19]. It causes neuronal apoptosis and in turn acute brain damage and is of critical importance in the development of neurodegenerative diseases.

Glutamate-induced oxidative stress is known to triggers neuronal death in primary neuronal cell cultures as well as cell lines [20]. Earlier studies shown that flavonoids and catechins with strong antioxidant activity protected glutamate-induced neuronal death [21]. These results indicate that oxidative stress induced by glutamate causes HT22 cell apoptosis, and this apoptosis is effectively suppressed by chemical compounds including flavonoids and polyphenols [22,23,24]. Plant extracts containing these compounds have antioxidant properties and are known to effectively suppress neuronal apoptosis caused by glutamate [25,26]. In this study, we used mouse-derived HT22 nerve cells to study the neuroprotective effect of catechins against glutamate-induced cell apoptosis and to decipher the neuroprotective mechanism of action [27]. These cells are widely used to study neuroprotection against glutamate damage. The mechanism of toxic response in HT22 cells was owing to the absence of functional anisotropic glutamate receptor, such as experimental model cells N-methyl-D-aspartate. Glutamate reduces the uptake of cystine via the glutamate/cystine antiporter, and low levels of cystine contribute to a decrease in glutathione synthesis [28]. Furthermore, low glutathione levels fail to activate the glutathione redox cycle, which involves glutathione peroxidase and glutathione reductase. This is classified as a non-receptor-mediated oxidative stress. Owing to the deficiency of glutathione, intracellular ROS cannot be effectively eliminated, which causes receptor-mediated oxidative stress [29].

It has already been reported that neuronal apoptosis induced by glutamate is mainly caused by oxidative stress, which is a result of excessive production of free radicals and low levels of antioxidants in cells [27]. In addition, previous studies have reported that phytochemical antioxidants inhibit neuronal cell death by preventing the accumulation of free radicals [30]. In this study, we confirmed that GCG, a catechin from green tea, has neuroprotective effects against glutamate-induced neuronal cell death. The results showed that GCG alone is not neurocytotoxic even at high concentrations of 100 and 200 μM, whereas EGCG was neurocytotoxic at similar concentrations. Furthermore, it was confirmed that the antioxidant and neuroprotective effects of GCG are equivalent to those of EGCG. In addition, it was found that the increased levels of ROS induced by glutamate were significantly reduced by GCG treatment, which was confirmed by studying the accumulation of ROS in cells. Thus, these results confirm that GCG, a potent antioxidant, exhibits neuroprotective effects by preventing the excessive accumulation of ROS in cells.

Oxidative stress progresses to downstream stages, including increased Ca^2+^ concentration through receptors, decreased mitochondrial membrane potential, mitochondrial malfunction, and eventually leads to cellular apoptosis [31]. We confirmed that GCG significantly reduced the amount of ROS and Ca^2+^ in HT22 cells using a specific dye. Based on these results, it could be concluded that GCG inhibits ROS influx through cellular receptors and also Ca^2+^ channels. Excess glutamate significantly increases the amount of ROS and Ca^2+^ and induces cell death. To investigate the effect of GCG on glutamate-induced apoptosis in HT22 cells, cells were treated with 5 mM glutamate in the presence or absence of 50 or 100 μM GCG for 12 h. We stained HT22 cells with Hoechst 33342 to confirm chromatin condensation, a hallmark of apoptosis [32]. As a result, chromatin condensation was observed in glutamate-treated HT22 cells, whereas GCG was shown to suppress this glutamate-induced effect. To confirm the neuroprotective mechanism of GCG against neuronal cell death, western blot analysis was performed to confirm the phosphorylation of ERK and JNK, and GCG was found to significantly reduce the phosphorylation of MAPK induced by glutamate.

N-acetylcysteine (NAC) is a precursor to the antioxidant glutathione modulates glutamatergic and inflammatory pathways, and emerging as treatment of neurodegenerative disorders. NAC acts as an antioxidant after converted to cysteine in the body, and reported to removes ROS. It is reported that NAC has a protective effect against oxidative damage by regulating the Nrf2/HO-1 signal activated by oxidative stress [33]. As a limitation of this study, further studies are required on the signaling mechanism of Nrf2/HO-1 [34] and the mechanism of mitochondrial protection, and external toxic brain injury models and treatment animal experiments are also needed [35].

In summary, we confirmed that GCG exhibited antioxidant potential and inhibited neuronal apoptosis, ROS, and Ca^2+^ influx induced by glutamate without causing neurocytotoxicity [36]. When GCG was used in combination with glutamate, the ROS and Ca^2+^ levels decreased along with cell apoptosis. Nuclear condensation was also found to be significantly reduced. These results showed that GCG protected against neuronal apoptosis by inhibiting ROS, Ca^2+^ influx, nuclear condensation, and phosphorylation of ERK and JNK.

## 4. Materials and Methods

### 4.1. Antioxidant and Neuroprotective Effects of Catechins

The five catechins (EGCG, GCG, GC, ECG, and EC) used in this study were purchased from Sigma-Aldrich (Saint Louis, MI, USA). Catechins used in cell experiments were dissolved in dimethyl sulfoxide (DMSO, Sigma-Aldrich) to obtain a final concentration of 100 mM. Cells were diluted in Dulbecco’ s Modified Eagle Medium (DMEM) medium to limit DMSO to a concentration of 0.5% (*v/v*) or less. DPPH was purchased from Sigma-Aldrich and Pierce™ BCA protein assay kit used in this experiment was obtained from Thermo Scientific (Rockford, IL, USA). Fetal bovine serum (FBS), penicillin (100 IU/mL), streptomycin (100 μg/mL), DMEM, and trypsin-ethylenediaminotetraacetic acid were purchased from Gibco (Waltham, MA, USA), and Dulbecco’s phosphate-buffered saline (DPBS) was purchased from WELGENE (Korea). EZ-CYTOX (Cell Viability, Proliferation & Cytotoxicity Assay Kit) was purchased from DoGenbio (Seoul, Korea). DCFDA and Hoechst 33342 were purchased from Sigma-Aldrich. Antibodies (p-p38, p38, p-JNK, JNK, p-ERK, ERK, GAPDH, and goat anti-rabbit IgG secondary antibody) were purchased from Cell Signaling Technology (Danvers, MA, USA). The instruments used in this study were a microplate reader (Molecular Devices, San Jose, CA, USA), inverted fluorescence microscope OLYMPUS 1X50 (Waltham, HA, USA), and Fusion Solo (Vilber Lourmat, Paris, France).

### 4.2. Evaluation of DPPH Scavenging Ability of Catechins

One hundred microliters of catechin solutions (3.15, 6.25, 12.5, 25, 50, 100, and 200 μM) were dissolved in DMSO and mixed with 100 μL of DPPH solution in a 96-well plate and blocked in the room. Absorbance was measured at 550 nm using EMax (Molecular Devices, Sunnyvale, CA, USA). DPPH free radical scavenging activity was expressed as a percentage (%) based on the experimental method described by Hatano et al. [37].

### 4.3. Neuroprotective Effect of Catechins against Glutamate-Induced Excitotoxicity

#### 4.3.1. Evaluation of Cell Viability

In this experiment, the mouse hippocampus-derived HT22 cell line was used. The cells were treated with various concentrations of catechins (3.125, 6.25, 12.5, 25, 50, 100, and 200 μM) to determine neurocytotoxicity. Cell viability was confirmed using EZ-CYTOX [38]. Cells were cultured in a 96-well plate at a density of 1 × 10^4^ cells/well in a volume of 100 μL/well and incubated for 24 h. Cells were treated with GCG, EGCG, GC, and ECG and cultured for 24 h. The control was media containing 0.5% (*v/v*) DMSO.

#### 4.3.2. Evaluation of Neuroprotective Effects of Catechins in HT22 Cells against Glutamate Excitotoxicity

The mouse hippocampal-derived HT22 cell line was used and the neuroprotective effect of catechins against glutamate excitotoxicity in the HT22 cell line was assessed [39]. HT22 cells (2 × 10^5^ cells/well) were inoculated into DMEM medium containing 10% heat-inactivated FBS, penicillin G (100 IU/mL), and streptomycin (100 μg/mL). After culturing for 24 h, catechins were added in different concentrations (3.125, 6.25, 12.5, 25, 50, 100, and 200 μM) followed by addition of 5 mM glutamate the cells were incubated for 12 h in a 5% CO_2_ incubator. Measurements were performed using the EZ-CYTOX method.

#### 4.3.3. Determination of ROS Levels

The effect of GCG on ROS production induced in HT22 cells by glutamate was determined using 2′,7′-dichlorofluorescin diacetate (DCFDA) staining [40]. HT22 cells were treated with GCG (50 or 100 μM) and 5 mM glutamate for 8 h and washed with PBS. Then, the cells were treated with Hanks’ balanced salt solution containing 10 μM DCFDA in the dark for 30 min. The amount of ROS formed in the cells were measured using a photometer (excitation wavelength: 485 nm; emission wavelength: 535 nm).

#### 4.3.4. Measurement of Ca^2+^ Levels

HT22 cells were treated with 50 μM glutamate and 100 μM GCG for 8 h, and then stained with Fluo-4, a fluorescent Ca^2+^ indicator. HT22 cells (2 × 10^5^ cells/well) were treated with GCG, 5 mM glutamate, and Fluo-4 reagent in a 6-well plate in the dark for 8 h. Cells were photographed using a light microscope.

#### 4.3.5. Evaluation of Nuclear Condensation Using Hoechst 33342 Reagent

The changes in nuclear morphology of HT22 cells due to glutamate were observed using Hoechst 33342 staining [41]. HT22 cells were cultured at 2 × 10^5^ cells/well in a 6-well plate, treated with 5 mM of GCG and glutamate for 8 h, and then treated with Hoechst 33342 reagent for 10 min. The cells were then photographed using an optical microscope.

#### 4.3.6. Protein Separation (Preparation of Whole-Cell Lysates)

We treated HT22 cells with 5 mM glutamate and 50 or 100 μM GCG for 8 h and confirmed MAPK phosphorylation using western blotting [42]. To determine the degree of apoptosis induction, HT22 cells grown in medium supplemented with 5 mM glutamate and GCG (50 or 100 μM) were harvested after centrifugation in a 60-mm culture dish and suspended in DPBS. The suspension was prepared in 1.5-mL Eppendorf tubes. After centrifugation at 4 °C and 5000 rpm for 90 s, the supernatant was removed, and the remaining cells were lysed with lx protease inhibition cocktail (Roche, Penzberg, Germany) and 1 mM phenylmethylsulfonyl fluoride buffer. The treated cells were centrifuged at 12,000 rpm for 20 min, and the protein supernatant was used for experiments. The concentration of each protein was determined using a BCA Protein Assay kit (Sigma-Aldrich).

#### 4.3.7. SDS-Polyacrylamide Gel Electrophoresis and Western Blot Analysis

After treatment with 5 mM glutamate for 8 h, HT22 cells were harvested and lysed using RIPA buffer (Cell Signaling, Danvers, MA, USA). They were separated using SDS-polyacrylamide gel electrophoresis and electroblotted with Immobilon^®^-PSQ PVDF transfer membrane (Millipore, Billerica, MA, USA). The membranes were then washed with PBS-T (phosphate buffered saline with Tween 20) and incubated with 5% skim milk at room temperature for 1 h to block nonspecific proteins. Each antibody was diluted (1:1000) with 5% skim milk and the reaction was carried out for 1 h. After washing with PBS-T, the secondary antibody was diluted (1:2000) with 5% skim milk and the secondary antibody reaction was induced at room temperature for 1 h. The amount of specific protein was then analyzed using LAS-4000 (Tokyo, Japan) and Enhanced Chemiluminescence Advance Western Blotting Detection Reagents (Waukesha, WI, USA). Western blot analysis was performed using p-p38, p38, p-JNK, JNK, p-ERK, ERK, GAPDH, and goat anti-rabbit IgG secondary antibody (Cell Signaling Technology, Beverly, MA, USA).

### 4.4. Statistics

All results are expressed as means  ±  S.E.M. The results were analyzed by one-way analysis of variance (ANOVA), followed by Tukey’s post hoc test. Differences were considered statistically significant at *p* < 0.05, 0.01 and 0.001.

## Figures and Tables

**Table 1:** Effect of gallocatechin gallate against glutamate-induced apoptosis in HT22 cells.

	Relative Protection (%)	
Concentration (μM)	GCG	5 mM Glu + GCG
0	100.00 ± 2.81	32.16 ± 3.01
3.125	95.54 ± 4.21	34.04 ± 4.53
6.25	95.87 ± 3.89	37.77 ± 3.21
12.5	96.80 ± 3.28	33.14 ± 4.47
25	94.62 ± 4.52	30.21 ± 4.25
50	94.87 ± 2.31	52.03 ± 2.31
100	96.57 ± 3.57	96.80 ± 3.57
200	94.67 ± 4.57	94.18 ± 2.57

## Data Availability

The data presented in this study are available on request from the corresponding author.

## Sample Availability

Samples of the compounds are or are not available from the authors.
